# RENATA study—Latin American prospective experience: clinical outcome of patients treated with palbociclib in hormone receptor-positive metastatic breast cancer—real-world use

**DOI:** 10.3332/ecancer.2020.1058

**Published:** 2020-06-17

**Authors:** Fernando Petracci, Gonzalo Gomez Abuin, Alejandra Pini, Matías Chacón

**Affiliations:** 1 Breast Cancer Department, Instituto Alexander Fleming and Sanatorio Las Lomas, Buenos Aires 1428, Argentina; 2 Oncology Service, Hospital Alemán, Buenos Aires 1428, Argentina; 3 Oncology Service, Hospital Militar Central and Sanatorio Las Lomas, Buenos Aires 1428, Argentina; 4 Oncology Service Chair, Instituto Alexander Fleming, Buenos Aires 1642, Argentina

**Keywords:** hormone receptor-positive, advanced breast cancer, Palbociclib, real-world, progression-free survival, overall survival

## Abstract

**Background:**

In hormone receptor-positive, HER-2 negative (HR+/HER2−) advanced breast cancer (ABC) endocrine therapy (ET) plus cyclin-dependent kinase 4/6 inhibitors (CDK4/6i) in first and second line improved progression-free survival (PFS), overall response rate (ORR) and clinical benefit rate (CB) without deterioration in quality of life compared with ET alone. In addition, recent data showed improvement in overall survival (OS) for premenopausal women in first line setting and for different subgroups of patients in second line. Since 2015, in Argentina, the combination of ET with CDK4/6i is a standard of care in HR+/HER2− ABC.

**Methods:**

We carried out a prospective analysis of real-world use of palbociclib with ET in HR+/HER2− ABC patients who received treatment between October 2015 and August 2019 in two private institutes from Buenos Aires, Argentina. The aims of the study were to determine efficacy and safety of patients treated with ET and palbociclib, describe patient profile and treatment strategy beyond progression.

**Results:**

One-hundred and twenty-eight patients were included in the final analysis. Main baseline characteristics include, median age 57 years, 20% were premenopausal women, 44% had visceral metastasis and 26% bone only disease. More than half of patients had two or more metastatic sites, 44.4% had performance status 1, and most of them (59.4%) were treated with palbociclib in first-line setting. Palbociclib was preferentially associated with aromatase inhibitors in 63.9% of patients, and with fulvestrant in the remaining. All premenopausal women received ovarian suppression or ovarian ablation (OS/OA). The median PFS was 36.7 months in first line and 24.2 months in second line. The ORR was 45.3% and 25.0% in first and second line, respectively. The median OS in the entire population was not reached. Half of patients did not require dose interruption and/or delay, dose reduction was required in 15% of patients and almost no patients required drug discontinuation (2.0%). With regard to safety, 55% of patients developed grade 3–4 adverse events, 20% neutropenia grade 3–4, and 7% febrile neutropenia. Infections were presented in one out of three patients, mostly uncomplicated.

**Conclusions:**

This is the first prospective evidence of real-world use of palbociclib in a Latin American population. We found similar outcomes to the PALOMA-2 and PALOMA-3 randomised trials and Real-World Data already published, with lower incidence of side effects and treatment discontinuation, but with higher incidence of febrile neutropenia.

## Background

As in many other countries breast cancer is the most frequent cancer in women in Argentina, with more than 21.000 new cases per year [1]. Worldwide, HR+/HER2- breast cancer is the most common subtype in advanced setting. For this scenario, CDK4/6i with ET is the new standard of care in first and second line based on higher rate of responses, longer progression-free survival (PFS) and overall survival (OS). In Argentina, palbociclib was approved in October 2015 by the government regulatory agency in first and second line in combination with aromatase inhibitors or fulvestrant.

The present study assessed the real-world effectiveness and safety of palbociclib combined with endocrine therapy (ET) for patients with HR+/HER2− advanced breast cancer (ABC) treated in two private institutes in Buenos Aires.

## Methods

### Study design

This prospective study was designed in 2015 when palbociclib was approved by the National Administration of Medicines—ANMAT (Administración Nacional de Medicamentos, Alimentos y Tecnología Médica). The aim of the study was to evaluate the clinical benefit, side effects and long-term survival of patients with HR+/HER2− ABC treated with palbociclib plus ET in different lines of treatment between October 2015 and August 2019. Inclusion criteria: pre and postmenopausal women, men, Oestrogen Receptor positive (defined by ER expression ≥1% of tumour cell by immunohistochemistry, IHC) and HER2 negative (by IHC and/or amplification assay) in primary tumour or metastatic site after biopsy, that have received at least one cycle of palbociclib in advanced setting.

### Data collection

All data were collected from original medical records from baseline to last visit or death. Data included: demographic information, age at first diagnosis and age at the beginning of treatment with palbociclib, clinical characteristics and performance status by Eastern Cooperative Oncology Group scale (ECOG).

***Treatment-related data:*** loco-regional and neo/adjuvant systemic treatment, number and type of treatments in advance setting before palbociclib, type of treatment beyond palbociclib progression, treatment strategy in premenopausal women (ovarian suppression / ovarian ablation, OS/OA), palliative radiation therapy before or during palbociclib treatment and partner of palbociclib in different lines.

***Metastatic data at the beginning of palbociclib:*** ‘de novo’ metastatic disease, site of metastases (bone, soft tissue, visceral, visceral and bone, central nervous system-CNS with or without other site), and metastatic site at palbociclib progression.

***Patients predisposition:*** side effects by frequency and grade (NCI-CTCAE version 4.0), starting dose and number of patients with dose interruption, delay, reduction or treatment discontinuation.

### Objectives

***Treatment benefit:*** Overall response rate (ORR) by medical consideration (complete response—CR and partial response*—*PR), stable disease (ED) at least ≥ 6 months without progression, and Clinical Benefit (CB: CR + PR + ED) in the overall population and by line of treatment, and symptomatic improvement in patients with ECOG ≥ 1 at the beginning of palbociclib.

***Patients outcome:*** PFS in first and second line and more (months from the date of therapy began to the date of progression as determined by treating physician or the date of patient death or last follow-up). OS by line and type of ET (months from the beginning of palbociclib to death or last follow-up) and OS beyond progression of palbociclib (OS* post-palbo*, months from the date therapy finished to death or last follow-up).

### Statistical methods

SPSS v26 software was used for statistical analysis. Demographic, clinical characteristics and side effects were analysed using descriptive statistics (count, percentages and median/range). Kaplan–Meier test was used to determine the median PFS and OS in the entire population and subgroups. Log rank test was used for comparisons of PFS among different subgroups. All statistical tests were two- sided, and the significance level was 0.05.

## Results

### Patient demographics

A total of 134 patients were identified since October 2015, six of whom were excluded because they were treated with ribociclib, were included 1 man, 4 patients with BRCA1/2 germline mutation, 1 patient with CHEK2 somatic mutation, 8 patients previously treated with everolimus and 3 patients with visceral crisis at the discretion of the treating physician. Strikingly, 20.7% of patients were diagnosed with ‘de novo’ metastatic disease, 48% of them with bone only disease and 22% with visceral involvement.

Regarding systemic treatment in primary diagnosis, 52% performed neoadjuvant and/or adjuvant CT, most of them with anthracyclines plus taxanes (51.3%), Cyclophosphamide, Methotrexate, Fluorouracil (CMF) (23.7%), and anthracyclines alone (22.4%). More than 90% of patients performed adjuvant ET, most of them with tamoxifene (82%), follow by switch strategy (A.I. after tamoxifene), and only 3% were treated with A.I. alone. Twenty-three premenopausal women developed amenorrhea after adjuvant systemic treatment, and nine patients were treated with OS/OA therapy.

Median follow-up since primary diagnosis was 99.4 months (95%CI 85.2–124.7) and median distant recurrence free survival was 73.3 months (95%CI 49.2–364.8).

At the beginning of treatment with palbociclib, median age was 57 years (range: 29–84). Visceral metastasis (29%) and bone only metastasis (26.6%) were the most frequent metastatic sites follow by soft tissue metastasis (skin, lymph nodes, pleura and peritoneal cavities). Half of the patients had two or more metastatic sites. Most of the patients were ECOG performance status 0–1 but 16% were 2 or 3, including three patients (2.6%) with visceral crisis criteria unfit for CT (one patient with CNS and meningeal involvement, one patient with liver dysfunction and one patient with unavailable data). More than half of the patients (55.5%) were naïve for ABC treatment, 23% received at least one line of ET plus CT and 20% received at least one line of CT or ET. About 30% of the patients underwent palliative radiation therapy (RT), most of them with symptomatic bone lesions. Palliative RT was done before start palbociclib in the majority of patients, or during week off in the minority. Premenopausal women (26 patients) performed OS/OA as recommended by international guidelines, of them 92.3% underwent bilateral salpingo-oophorectomy and only in 7.6% of them LHRH agonist was recommended.

### Treatment and outcome

Palbociclib was indicated in first line in 59.4% of patients, 15% in second line and 26% in third or more lines. In 61.7% of patients, letrozole was the partner of palbociclib follow by fulvestrant in 35.9% of patients. In a small number of patients, palbociclib was combined with exemestane or anastrozole. Since October 2015 until last follow-up, 18 patients died and 27 patients lost during follow-up.

Of total, 109 of patients were available to assess the rate response. The ORR was 45.8%, 3.6% of patients achieved CR and 42.2% PR. The Overall CB was 82.5%, higher in first line than in second line, 85.9% and 37.5%, respectively.

For those symptomatic patients at the beginning of palbociclib treatment, 65.4% improved their ECOG performance status with a small difference between first and second line, 63.3% and 50.0%, respectively.

Until August 2019, 57 patients developed disease progression during palbociclib and two patients discontinued treatment due to haematological toxicity. Visceral progression (64.4%) was the most frequent site of progression followed by visceral and bones (16.0%) and bone only disease (14.2%). CNS was infrequent site of progression (5.3%). Strikingly, 14 patients (24.5%) with visceral compromise were considered as visceral crisis by oncologist criteria. Chemotherapy was the treatment of choice after palbociclib progression in 64.0% of patients, being the most frequent regimens based on taxanes (with or without bevacizumab) and capecitabine. Additionally, ET was indicated in 24.0% of patients, with exemestane plus everolimus as most frequent combination. Only 10.0% of patients were unfit to CT (endocrine-resistance) in which palliative care was recommended (Table 1).

**Figure 1.**
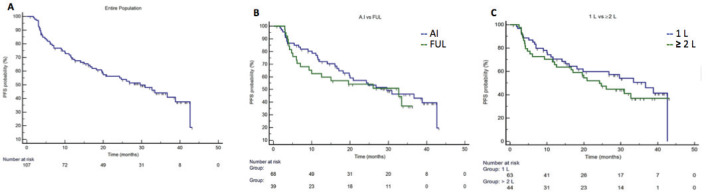
Progression-fee Survival. Panel A. Entire population, median PFS was 29.6 months (95%CI 19.5–38.8). Panel B. Median PFS in palbociclib with A.I. group was 29.6 months (95%CI 18.1–42.6), and in the palbociclib with fulvestrant subgroup was 32.7 months (95%CI 9.3–33.4). Panel C. Median PFS in first line setting was 36.7 months (95%CI 18.1–42.6) and in second line and more was 24.2 months (95%CI 12.0–32.7).

**Figure 2.**
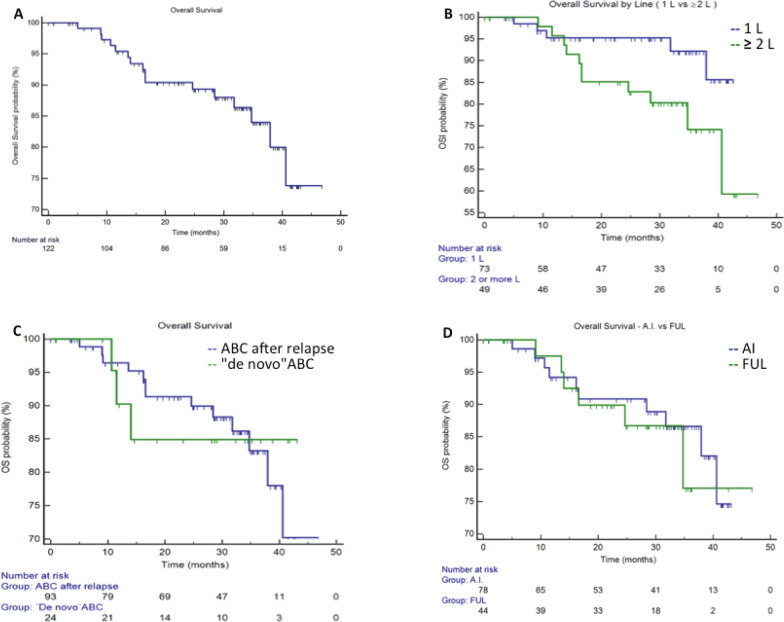
Overall Survival. Panel A. Entire population, median OS was not reached. Panel B. Median OS in first line and ≥2 lines was not reached.

Median PFS in the entire population was 29.6 months (95%CI 19.5–38.8), in first line setting the median PFS was 36.7 months (95%CI 18.1–42.6) and in second or more lines was 24.2 months (95%CI 12.0-32.7) (Figure 1). Median PFS in A.I. based treatment was 29.6 months (95%CI 18.1–42.6), and in fulvestrant subgroup was 32.7 months (CI95% 9.3–33.4). The median OS in the entire population since starting palbociclib was not reached. At 36 months of follow-up, there were 92.8% of patients alive in the first line setting and 74.0% in those patients treated in second line and more. (Figure 2). The median OS post-palbo was 15.6 months (95%CI 4.8–26.3).

### Safety

The starting dose was 125 mg daily (day 1 to 21 every 28 days) in 96.0% of patients, with a scanty number of patients starting with 100 mg or 75 mg due to age (>75-year old), co-morbidities or bone marrow insufficiency as according to treating physician decision. Haematological toxicity was the main reason for dose delay or interruption in 46% of patients, the rate of dose reduction was 15.2%, and almost none of patients required drug discontinuation (2.0%). During the first year of institutional experience, granulocyte-colony stimulating factors were indicated in 10% of patients.

Data from 100 patients were available to assess toxicity, 55.0% of them developed grade 3 and 4 adverse events, 82% neutropenia (any grade), 20% grade 3 and 4 neutropenia and 7.6% with febrile neutropenia (at least one event). One out of three patients (27.5%) developed at least one event of infection during treatment, most of that patients with not complicated infections.

Upper air tract infections in 8 patients, urinary tract infection in 6 patients, dermatologic or mucosal zoster virus reactivation in 5 patients, 3 patients with pneumonia (one of them with abscessed pneumonia) and acute colitis in 1 patient. Uncommon side effects (less than 2 patients, most of them G1) were: pulmonary embolism, xerostomia, cutaneous rash, hyperglycaemia, femoral osteonecrosis, acute blepharitis and hand-foot syndrome (Table 2).

## Discussion

The aim of this prospective study was to evaluate the real-world use of palbociclib in combination with ET for HR+/HER2− ABC. Few real-world evidence studies of palbociclib used in daily clinical practice have been published identifying clinical benefit, patient profile and sequencing of treatment, with even less evidence of use of palbociclib in Latin American patients. In RENATA study, we included 60% of patients treated with palbociclib in first line and 40% in second or more lines, and palbociclib was combined with letrozole in 62% of patients and in 36% with fulvestrant. As the PALOMA-3 trial (21%) we treated 20% premenopausal women with palbociclib, all of them underwent to OS/OA. In the prospective POLARIS study, palbociclib was used in first line in 70% of patients, in combination with letrozole/anastrazole, fulvestrant and exemestane in 57%, 39% and 4%, respectively [5]. Regarding patient profile, in our study, 29% had visceral metastasis and 27% bone only disease comparing with 49% and 38% in PALOMA-2, and 60% and 25% in PALOMA-3, respectively [3, 4]. Important to mention, 55% of patients were naïve for advanced treatment, and 20% were ‘de novo’ stage 4, lower than 37% of ‘de novo’ stage 4 in PALOMA-2 and 30% in PALOMA-3 study. The ORR in RENATA study was 45% in first line (with 3.6% of CR), and 25% in second line (without CR), similar rates than PALOMA-2 (ORR: 42%) and PALOMA-3 (ORR:19%, without CR). The overall CB was 82%, 86% in first line (85% in PALOMA-2) and 38% in second line (67% in PALOMA-3). Regarding performance status, 65% of patients reported symptomatic improvement since starting with palbociclib. The median PFS in first line setting was 36.7 months longer than median PFS reported in PALOMA-2 study (24.8 months) [3].

Regarding grade 3 and 4 neutropenia, we observed three-fold lower incidence in our study (20%) than the results of the PALOMA-2 and PALOMA-3 (66% and 65%, respectively). Unlike the published data, we found higher incidence of febrile neutropenia (7.6%) compared to 1.3% in PALOMA-2 and only 1 patient in PALOMA-3. Looking for predictors of febrile neutropenia, we identified six out of eight patients over 65-year old, most of them with bone involvement, heavily pretreated, and palliative radiation therapy history. Patients treated with CDK4/6 inhibitors are susceptible to viral or bacterial infections, the rate of infections in PALOMA-3 was 42% and were not reported in PALOMA-2. In our experience, 27.5% developed at least one episode of infection during treatment, most of them uncomplicated.

The incidence of dose reduction in our experience was 15%, lower than randomised trials PALOMA-2 (36%) and PALOMA-3 (34%), and similar to dose reduction rate with A.I. combination (20%) or with fulvestrant (14%) in the retrospective U.S. real-world practice IRIS study [6]. Regarding patient predisposition, 50% of our patients needed at least one dose interruption and/or delay during treatment, a bit lower than PALOMA-2 (67%) and similar to PALOMA-3 (54%). We found lower incidence of treatment discontinuation (2.0%) compared to PALOMA-2 (4%) and PALOMA-3 (10%) trials. As many prospective and retrospective studies, in our experience haematological toxicity was the main reason for dose modification and treatment discontinuation.

The pooled analysis of the 3 PALOMA trials performed by Dieras *et al* [8] that represents the largest and most comprehensive analysis of the long-term safety of palbociclib, found that neutropenia (any grade, 80.6%) and infections (54.7%) were the most common adverse events in patients treated with palbociclib plus ET. In this report, the majority of patients (63%) did not require a dose reduction and permanent discontinuation due to adverse events was necessary in 8.3% of patientes.

Visceral involvement was the most common site of progression during palbociclib (64%), with 24.5% of patients developing visceral crisis, because of that, CT was the first treatment of choice beyond progression. Only 24% of patients continued with ET after palbocilib progression, most of them with exemestane plus everolimus combination.

Recently, data from the Argentine subgroup of patients in the IRIS Study was published. The main differences between RENATA and IRIS studies are: a) our study collected data prospectively, IRIS study is a retrospective data review, b) we included three times more premenopausal women than IRIS study, 26 versus 9 patients, respectively, c) long-term overall survival data in the IRIS study was at 18 months, versus 36 months in our experience, d) IRIS included higher number of ‘de novo’ stage 4 disease (39% versus 20%), e) both studies included similar number of patients with visceral involvement (IRIS: 29% lung and 19% liver, and RENATA: 29% visceral and 15% visceral and bone compromise), f) identical number of patients treated with letrozol (RENATA: 62% and IRIS: 65%) and fulvestrant (RENATA: 36% and IRIS: 35%) and by last g) the RENATA study found lower ORR (CR: 4% and PR: 42%) than IRIS study (CR: 6% and PR: 60%) [7].

The main limitation of our study is related to the collection of data on clinical records, depending exclusively on the oncologist’s willingness to detail patient data, its treatment and clinical outcomes. Otherwise, its strength lies in its prospective character and long follow-up.

## Conclusion

The RENATA study is the first prospective experience utilising palbociclib in daily clinical practice in the Latin American population with HR+/HER2− advanced-stage breast cancer diagnosis. Our study showed similar results in ORR, CB and symptomatic improvement to PALOMA-2 and PALOMA-3 despite heterogeneous population, heavily pretreated in adjuvant setting, including patients with poor performance status, visceral crisis and pretreated with everolimus. Palbociclib in daily practice demonstrated lower incidence of dose interruption or delay, lower chances of dose reductions and discontinuation compared to data from pivotal trials previously published.

The RENATA study reinforces the use of palbociclib as a standard of care in HR+/HER2 metastatic breast cancer.

## Clinical practice highlights

First Latin American prospective study with palbociclib plus endocrine therapy,

The ORR in first line was 45% with a median PFS of 36.7 months,

The overall CB was 82%, with symptomatic improvement in 65% of patients,

In first line setting, 92% of patients were alive at 36 months of follow-up,

Lower incidence of dose reductions (15%), dose interruption and/or delay (50%) and discontinuation (2%),

Visceral progression was the most common site of progression (45%), forcing the use of CT as the first treatment strategy (64%) beyond palbociclib progression.

## Conflicts of interest

There are no conflicts of interest.

## Funding statement

This research did not receive any specific grant from funding agencies in the public, commercial or not-for-profit sectors.

## Ethics statement

All patients signed informed consent authorising the use of the data dumped in the electronic health record. Data collection was anonymous. Given the characteristic of the study, it was reviewed and authorised by the local institutional Ethics Committee.

## Figures and Tables

**Table 1.** Treatment beyond Palbociclib progression.

Treatment beyond Palbociclib progression (n:109 p)		
**(*n*: available)**	***n***	**%**
Progression sites (57) Visceral Visceral + bone Bone Bone + soft tissue Soft tissue CNS w/wo other Missed	26 9 8 7 3 3 1	45.6 15.7 14.0 12.2 5.2 5.2 1.7
Visceral crisis (57)	14	24.5
First Treatment beyond progression (50) Chemotherapy Taxanes ± bevacizumab Capecitabine Others Endocrine therapy Everolimus + exemestane Fulvestrant Exemestane Palliative care Investigational drug	32 14 12 6 12 9 2 1 5 1	64.0 28.0 24.0 12.0 24.0 18.0 4.0 2.0 10.0 2.0

**Table 2.** Specific toxicity with Palbociclib.

Specific Toxicity with Palbociclib (n:128 p)		%				
	***n* available**	**No**	**G1**	**G2**	**G3**	**G4**
Leukopenia	98	14.3	57.6	31.3	6.1	8.2
Anaemia	99	57.6	31.3	6.1	4.0	1.0
Thrombocytopenia	99	75.8	14.1	7.1	3.0	0
Fatigue	99	44.4	39.4	12.1	4.0	0
Headache	98	93.9	6.1	0	0	0
Alopecia	76	78.9	19.0	1.3	-	-
Nausea	99	86.9	11.1	2.0	0	0
Vomiting	99	94.0	4.0	2.0	0	0
Diarrhea	99	84.8	7.1	6.1	2.0	0
Constipation	100	90.0	7.0	3.0	0	0
Hot flushes	99	91.9	4.0	3.0	1.0	0
Arthralgia	99	69.7	25.3	4.0	1.0	0
Cough	97	93.8	5.2	1.0	0	0
Decreased appetite	98	91.8	8.2	0	0	0
Stomatitis	98	93.9	4.1	2.0	0	0
Mucositis	99	97.0	2.0	1.0	0	0
Dizziness	99	92.9	5.1	1.0	1.0	0
Abdominal pain	95	93.7	6.3	0	0	0
Pyrexia	94	95.7	4.3	0	0	0
Pruritus	92	95.7	4.3	0	0	0
Others *	88	91.0	9.0			

* Pulmonary embolism 1 p; xerostomy 1 p; cutaneous rash G1: 1 p, G2: 1 p; hyperglycemia G2: 1 p; femoral head osteonecrosis: 1 p; acute blepharitis G1: 1p; hand-foot syndrome G1: 1p.
